# Methodologies for the collection of parameters to estimate dust/soil ingestion for young children

**DOI:** 10.3389/fpubh.2024.1357346

**Published:** 2024-06-26

**Authors:** Alesia Ferguson, Foluke Adelabu, Helena Solo-Gabriele, Emmanuel Obeng-Gyasi, Cristina Fayad-Martinez, Maribeth Gidley, Jenna Honan, Olusola O. Ogunseye, Paloma I. Beamer

**Affiliations:** ^1^Department of Built Environment, North Carolina Agricultural and Technical State University, Greensboro, NC, United States; ^2^Department of Chemical, Environmental, and Materials Engineering, University of Miami, Coral Gables, FL, United States; ^3^Department of Community, Environmental and Policy, Mel and Enid Zuckerman College of Public Health, University of Arizona, Tucson, AZ, United States

**Keywords:** soil ingestion, dust ingestion, MLATS, children’s activity patterns, exposure

## Abstract

**Background:**

Heavy metals, pesticides and a host of contaminants found in dust and soil pose a health risk to young children through ingestion. Dust/soil ingestion rates for young children can be estimated using micro-level activity time series (MLATS) as model inputs. MLATS allow for the generation of frequency and duration of children’s contact activities, along with sequential contact patterns. Models using MLATS consider contact types, and transfer dynamics to assign mechanisms of contact and appropriate exposure factors for cumulative estimates of ingestion rates.

**Objective:**

The objective of this study is to describe field implementation, data needs, advanced field collection, laboratory methodologies, and challenges for integrating into and updating a previously validated physical-stochastic MLATS-based model framework called the Child-Specific Aggregate Cumulative Human Exposure and Dose (CACHED) model. The manuscript focuses on describing the methods implemented in the current study.

**Methods:**

This current multidisciplinary study (Dust Ingestion childRen sTudy [DIRT]) was implemented across three US regions: Tucson, Arizona; Miami, Florida and Greensboro, North Carolina. Four hundred and fifty participants were recruited between August 2021 to June 2023 to complete a 4-part household survey, of which 100 also participated in a field study.

**Discussion:**

The field study focused on videotaping children’s natural play using advanced unattended 360^°^ cameras mounted for participants’ tracking and ultimately conversion to MLATS. Additionally, children’s hand rinses were collected before and after recording, along with indoor dust and outdoor soil, followed by advanced mass analysis. The gathered data will be used to quantify dust/soil ingestion by region, sociodemographic variables, age groups (from 6 months to 6 years), and other variables for indoor/outdoor settings within an adapted version of the CACHED model framework.

**Significance:**

New innovative approaches for the estimation of dust/soil ingestion rates can potentially improve modeling and quantification of children’s risks to contaminants from dust exposure.

## Introduction

Children are exposed to environmental contaminants found in soil and dust in a number of settings including homes, schools, playgrounds, and beaches (1). Other indoor and outdoor environments (e.g., malls, gyms) may also play a smaller role in exposure. The contribution of each setting to overall exposure is dependent on time spent in a certain environment and the specific activities that contribute to dust and/or soil ingestion. Dust is found in the indoor environment while soil is found in the outdoor environment, including outdoor attached spaces (e.g., patios) (2, 3). Soil that has migrated or been tracked into the home is also considered as dust. Contaminants (e.g., heavy metals, dioxins, pesticides, allergens) are found in dust and soil based on numerous natural and manmade pathways, such as deterioration of building materials, use of various products in the home, migration of metals from mine tailings, wind dynamics and water runoff from industrial processes and farming (4–7). Dust is a heterogeneous mixture of soil from outdoors, lint, skin particles, organic fibers, food debris, etc. (8), and can be a repository for various harmful chemicals. Indoor dust can also play a role in inhalation exposure to viral agents such as COVID 19, especially during resuspension, and can in fact be used as a marker for tracking viral spread (9–14).

**Figure 1:**
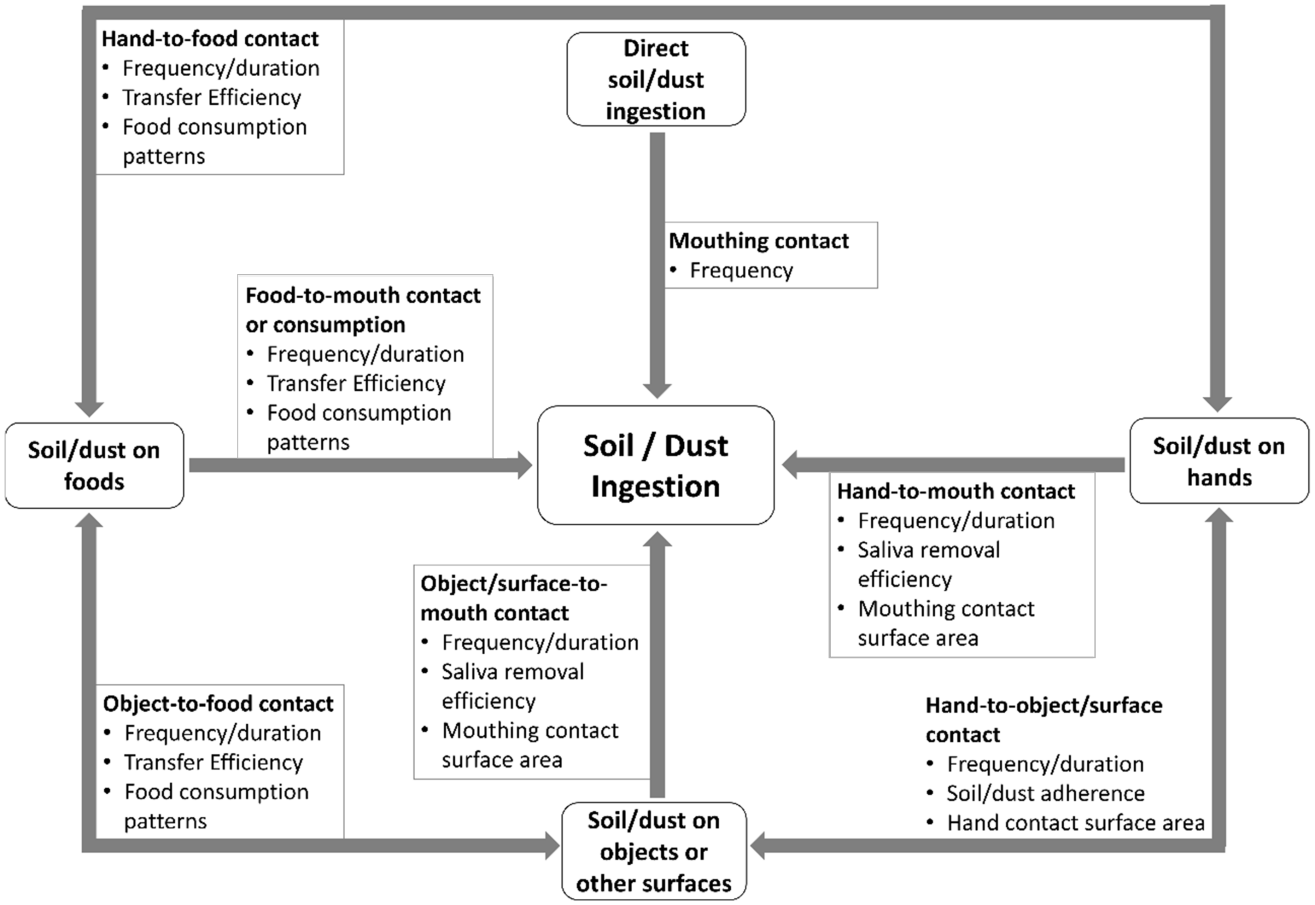
Four primary exposure pathways exist that lead to non-dietary ingestion of dust/soil or children. Each pathway requires consideration of quantified activity patterns and relevant exposure factors as shown. Not shown are other model parameters (e.g., dust-to-skin transfer) needed to estimate how for example dust adheres to objects and hands before transfer to the mouth.

Children are exposed to dust and soil ingestion primarily via four pathways: (1) dust/soil found on objects/surfaces placed in the mouth, (2) dust/soil placed directly into the mouth, (3) dust/soil found on food placed in the mouth, and (4) dust/soil found on hands (or other parts of the body) placed in the mouth after contact with dust/soil (Figure 1). Some inhaled dust can also be swallowed. It is critical to investigate various exposure pathways and understand how each contributes to total dust/soil ingestion rates. This allows for the development of more targeted prevention strategies to reduce contaminant exposures.

Dust and soil ingestion rates are used by exposure scientists and risk assessors in algorithms to estimate the amount of a contaminant ingested daily and over a lifetime, which can then be related to the toxic nature of the contaminant and ultimately to adverse health outcomes (15–24). This allows for decisions to be made on the potential need for prevention strategies if exposures are deemed unacceptable, such as dust/soil cleanup approaches in and around homes and at various sites (e.g., Superfund sites), restrictive regulations on the composition of consumer products brought in a home, and outreach to alter human behavior to reduce exposures, such as removal of shoes at doors to reduce track-in of soils. Higher soil ingestion rates have been documented as a key parameter responsible for influencing exposure to harmful contaminants for children (21), whereas a higher frequency of hand washing (i.e., behavioral activity) could potentially reduce the risk (25). In fact, exposure to heavy metals via hand/object-to-mouth intake pathways has been associated with the greatest health risk (21).

The US EPA estimates that children ingest approximately 60 to 100 mg/day of total dust and soil, and states this is based on limited data (26). Those dust/soil ingestion rates vary by children’s age and activities, housing characteristics and dynamics, dust loading rates, and environmental conditions (26). These estimates have a high degree of uncertainty due to variability in study methodologies and small datasets with limited statistical power to show significance in findings (20). Additionally, the dust/soil ingestion data collected to date from children lack representation across age groups, geographic regions, built environment and sociodemographic groups (27, 28).

The three approaches commonly used for dust/soil ingestion for children include: the tracer approach, the limited biokinetic model approach, and the activity modeling approach. The tracer approach traditionally involves the study of chemicals that are not metabolized in the body in a mass balance equation. Estimates of the total mass of dust/soil ingestion are based on the consumption (input) of known or measured tracer element concentrations, such as those measured in nearby soil samples, that are then compared to levels measured in feces, food and/or urine (output) after accounting for trace elements ingested from medicines, supplements and sometimes from toothpaste (29). Calabrese et al. (30) used eight tracer elements (e.g., aluminum and silicon) and a mass balance approach, that also considered food and medicine sources, to obtain soil ingestion rates of 9 to 96 mg/day for 64 children, ages 1 to 4 years. Similarly, Yang et al. (21) recently estimated median soil ingestion rates of 148 mg/day using the tracer method to look at metal exposure for 66 children living near e-waste recycling sites (21). However, the most important aspect of the tracer method is the use of a commonly found tracer with good recovery rates that can be effectively reconstructed in an exposure-biomarker model, which can be challenging (31). In particular, the pharmacokinetic knowledge of a tracer compound can be incomplete and vary across individuals and age groups leading to over or underestimates of soil/dust ingestion rates (32). Other challenges include the ability to distinguish routes of exposures, or particular sources like food or toothpaste, and the ingestion of soil versus dust linked to time spent in indoor versus outdoor environments (24).

The biokinetic model approach reflects the general concept in aggregate exposure assessments since it recognizes that exposures from different pathways do not occur as separate events. In this approach, actual measurements of a biomarker (e.g., toxicant levels in blood or urine-typically lead) are compared to predictions from a biokinetic model that incorporates toxicant exposures from food, water, air, soil, and dust via dermal, inhalation, and ingestion routes. Von Lindern et al. (22) used a biokinetic model approach to estimate dust/soil ingestion rates of 51 to 96 mg/day via blood lead biomonitoring for children 1 to 9 years of age near a superfund site in Idaho.

For the estimation of dust/soil ingestion rates that involves complex pathways and loading dynamics, activity pattern collection and analysis, the modeling approach is recognized as a valuable and often critical tool in the field (18, 33). The activity modeling approach considers the frequency and duration of children’s activities that may expose them to dust and soil, estimates ingestion for each contact given the activity type, and quantifies dust and soil uptake from the accumulation of contact events. A number of studies have used this approach by collecting and analyzing children’s activity patterns for use in modeling across some indoor and outdoor environments (33, 34). Refined modeled estimates of dust and soil ingestion rates for children have been presented by Ozkaynak et al. (18), where authors were able to separately identify dust and soil ingestion rates by pathways and child activities using a previously developed EPA Stochastic Human Exposure and Dose Simulation (SHEDS) Model. Data sources for the SHEDS model included video data from previous studies on contact frequency and duration for children, along with mouth removal efficiencies, and surface areas of contact found in the literature, for example. Their model showed the mean rate for soil ingestion (children of 3 to 6 years) of 41 mg/day, a mean rate of 20 mg/day for hand-to-mouth dust ingestion, and a mean rate of 7 mg/day for object-to-mouth.

In 2021, EPA issued a request for proposals (RFP) to expand the data set of dust and soil ingestion rates specifically for children between the ages of 6 months to 6 years (35). An emphasis was placed on data that represent diverse geographic regions, built environments (urban, suburban, rural), and sociodemographic characteristics. This study was developed in response to the EPA RFP, with a goal to utilize a comprehensive modeling approach (i.e., activity modeling) to integrate new and existing data, and to measure distributions of dust/soil ingestion rates by region, sociodemographic variables, location, age groups, and other variables related to indoor/outdoor settings. This study focuses on describing all project methodologies with an emphasis on new or refined techniques from the exposure field for estimation of dust/soil ingestion rates. The results from this study will be presented in subsequent publications. Additionally, proposed work from other research teams, funded by the US Environmental Protection Agency, are summarized at the end of this article to provide comprehensive information about the state-of-the-art approaches for gathering and analyzing data.

## Methods

### Data collection

The purpose of the study was to increase the size of the datasets available (i.e., datasets of mouthing contact frequencies, surface and hands dust loadings, time spent), combine it with data from previous studies, to increase the sample size used in developing parameter distributions for model inputs, thereby decreasing uncertainty in risk estimates. This was accomplished by recruiting an equal proportion of participants among the US EPA recommended age groups (36) [6 months to <12 months, 1 year (12 months) to <2 years, 2 years to <3 years, 3 years to <6] and through using the videotaping/videotranslation and dust data collection from each participant’s home. Data were collected across three environmentally distinct regions: Tucson, Arizona; Miami, Florida and Greensboro, North Carolina, beginning in August 2021 through June 2023, with data analysis and model simulations continuing sometime after. The three regions are environmentally distinct based on temperature, rainfall and humidity: Tucson-Arizona is hot-desert/hot semi-arid, Greensboro, North Carolina is humid-subtropical, and Miami-Florida is considered tropical wet (37), which can for example affect child time spent outdoors playing, soil re-suspension and potentially dust levels indoors. A total of 450 participants (150 participants in each region) participated in a survey, and 100 out of the 450 also participated in the field sampling (videotaping and dust/soil collection). The Florida research team was responsible for the processing and analysis of all dust/soil samples collected across the regions. The Greensboro research team was responsible for videotapes processing and videotranslation to produce MLATS data. The Arizona research team was responsible for model development using data generated/analyzed by the Florida and Greensboro research teams.

### Model approach

The ultimate goal of the study is to estimate dust/soil ingestion for children between the ages of 6 months and 6 years using a micro-level activity time series (MLATS) model. In this study, MLATS were collected via newer videotaping and videotranslation methodologies. Videotranslation refers to the conversion of activity patterns from video footage to text files to be used in models. This research team, along with other teams, have used videotaping and videotranslation methods in the past to collect activity patterns for children, useful in exposure and risk estimates (38–42).

We have adapted our previously developed MLATS-based physical-stochastic modeling framework that utilizes a number of parameters to estimate non-dietary ingestion rates (43–45). These include time series of hand-to-mouth and object-to-mouth events by a child, dust and soil adherence/removal transfer rates, and dust and soil loadings on the hands and objects. The final model outputs are the temporally averaged or integrated mass of dust and soil ingested, based on the mouthing removal rate per contact event. The non-dietary ingestion model links to a dermal exposure model using the same related input modules, such as MLATS and exposure factors including mass loading. In this study, the dermal module provides estimates of soil/dust loading on the hands. Dermal simulations can be used to develop parametric or empirical distributions of this loading on the hands for use in the non-dietary ingestion module. Dermal exposure is a function of transfer (adherence/removal) factors, contaminant concentrations, fraction of surface area contacted, and other exposure factors (Equation 1). The interest in the dermal model is the loadings on the hands. Non-dietary ingestion uses similar inputs but requires a total surface area in contact in lieu of a unitless fraction surface area. Instead of accumulating mass per area over time, each exposure is assumed as a discrete event that occurs instantaneously upon contact. The ingestion rates can then be used in the intake rate (IR) term to calculate an exposure dose to a contaminant (Equation 2). The basic construct of these algorithm/equations are the backbone of the more complex model used in this study (i.e., CACHED) that utilizes sequential contact behaviors (time-series) (43). It must be mentioned that not all the contaminants ingested in the soil matrix are bioavailable, due to excretion and elimination processes (46).

Equation 1: Dust/Soil Dermal Uptake Algorithm (47, 48).


(1) $$\mathrm{Dder}=\frac{C\times SA\;X\;AF\;\mathrm{XABS}\times EF\times CF}{BW}$$

Where:

Dder = Dermal Absorbed Dose (mg/kg/day).

C = Contaminant Concentration (mg/kg).

SA = Surface Area Available for Contact (cm^2^/event).

AF = Soil-to-Skin-Adherence Factor (mg/cm^2^).

ABS = Absorption Factor (unitless).

EF = Exposure Factor (unitless).

CF = Conversion Factor (10^−6^ kg/mg).

BW = Body Weight (kg).

Equation 2: Dust/Soil Ingestion Algorithm (26, 49).


(2) $$\mathrm{Ding}=\frac{C\times IR\times EF\times CF}{BW}$$

Where:

Ding = Exposure Dose (mg/kg/day).

C = Contaminant Concentration (mg/kg).

IR = Intake Rate (mg/day).

EF = Exposure Factor (unitless).

CF = Conversion Factor (10^−6^ kg/mg).

BW = Body Weight (kg).

### Training

Training of personnel occurred at each study region for survey collection using Research Electronic Data Capture (REDCap) and for interacting with participants. REDCap is a secure and easy to use online data collection tool designed by a team at Vanderbilt University and used by many researchers nationally for survey collection and analysis (50). For the field study, lead researchers met in Greensboro during February 2021 for an in-person training on methodologies, and to complete a trial in-home field data collection. Lead researchers were then able to train and mentor their regional research teams. Written standard operating procedures and methodologies were created and used as a reference to routinely guide survey and field activities. Additionally, the project included a quality assurance official who conducted quarterly checks of adherence to procedures and completion of project documentation. Throughout the project, the team diligently met each month to discuss activities, tasks and project goals.

### Study recruitment

The universities used various strategies for recruitment, including assistance from community partners in the regions. The University of Miami, for example, used the Debbie Institute Mailman Center for Child Development, while the University of Arizona used El Rio Community Health Center, both of which have provided support in prior community-engaged research studies. All universities also conducted recruitment at various community locations and events, including at malls, museums, libraries, parks and (with permission) at daycare centers and health department clinics. Sampling was primarily by convenience with an effort to work with partners who had more access to minority/underserved populations (i.e., Black and Hispanic populations). Recruitment materials (including IRB approved flyers and postcards) were also posted on Facebook, Twitter, and YouTube with proactive postings to environmental advocacy and parenting sites. When in the field recruiting, or over the phone, researchers explained to parents the commitment requirements and value of the study. It was important to also express the low risks involved in participation. If parents showed interest, the recruitment team would follow up to schedule survey or videotaping times and days. During the time-periods heavily affected by the COVID-19 pandemic (for this study, January 2022 through January 2023), recruitment was exceptionally challenging, as participants had more safety concerns and reluctance to allow researchers in homes. Those who completed the dust loading survey received a $25 gift card. Participants and their children who additionally participated in the field study (video-recording and sample collection) received a second gift card valuing $100. Surveys, consent forms and outreach materials were translated into Spanish to also recruit Spanish-speaking participants. A family was able to complete the survey for up to four children in the family under the age of 6 years, and up to three children from a family could participate in the field-study.

### Human subject approval and subject protection

The study received Institutional Review Board (IRB) approval at North Carolina Agricultural and Technical State University (NCAT; protocol #21–0100). The University of Miami and University of Arizona agreed to have NCAT IRB as the designated IRB of record. All participants were consented before survey collection and field study participation. All research personnel were required to have human subjects training certification including modules that provide additional considerations needed when working with children. Given that data collected commenced during the COVID-19 pandemic, research personnel followed COVID mandates from the state and local officials during field studies and collection of survey data. Additionally, data generated during survey collection and household visits (i.e., field surveys) referenced child codes (i.e., identifiers), date and regions of data collection for purposes of protection, consolidation and analysis.

### Household dust survey

The survey was administered orally by researchers using an iPad, laptop, iPhone, or paper version in the field, in English and Spanish, following consenting. The survey took approximately 45 to 60 min to complete. Participants were able to complete the survey over the phone or in person with the researchers. The survey was programmed in the REDCap platform, allowing for easy analysis and transfer into statistical programs. All researchers in all regions used the same REDCap link to access the survey.

The household dust survey was divided into four distinct sections: family and child demographics, child health and activity, household routines, and an observational section. The child health-activity section requires the parent to recall some typical activities of the child that might affect ingestion exposures around the home such as tendency and frequency to mouth objects, suck fingers, use of a pacifier, handwashing frequency and time spent indoors versus outdoors. There was only one general health question on typical childhood illnesses (e.g., asthma). The household section looked at housing characteristics and house-cleaning and maintenance habits such as regularity of filter changes, type of ventilation and cooling systems, frequency of cleaning, wipe and vacuum methods used for floors, fans, and windowsills. Questions pertaining to specific variables that had previously been reported by other studies to be strongly associated with dust loading, such as age of home, and number of people in the home, were also included in the survey (51–55). The observational section looks at the layout of the home and yard, peeling paint, number of screens and location to certain contaminant and dust generating sources (e.g., roadway, gas stations). This section was completed only for those families participating in the field study component.

Survey data collected through this study will be analyzed using Classification and Regression Trees (CART). CART is a machine learning technique used for classification and prediction tasks useful for analyzing survey data with varied questions that might be related (56, 57). With the wealth of information and varied types of questions in the survey that are related to dust loadings in homes, behaviors of children and ultimately dust ingestion, this technique builds and evaluates a decision tree that describes relationships between variables and provides predictable outcomes. CART works by repeatedly partitioning data into smaller and smaller groups based on the values of the predictor variables until the resulting groups are homogeneous with respect to the outcome variable. The versatility afforded by a CART analysis to make decisions has been demonstrated in a variety of settings, such as seasonal ozone levels, screening indicators for COVID 19, and looking at social indicators in public health monitoring and intervention needs (58, 59). For this study, we predict that the CART analysis based on variables in the survey will show how and to what extent time spent outdoors for a child might be related to the size and location of the home, or how a parent’s race and income, and even the size of a home, might be related to the maintenance of home in terms of cleaning practices for the 450 participants. Additionally, we will be looking for relationships between survey responses and hand dust loadings we collect for the 100 participants that participate in the field study.

### Field study general process

**Figure 2:**
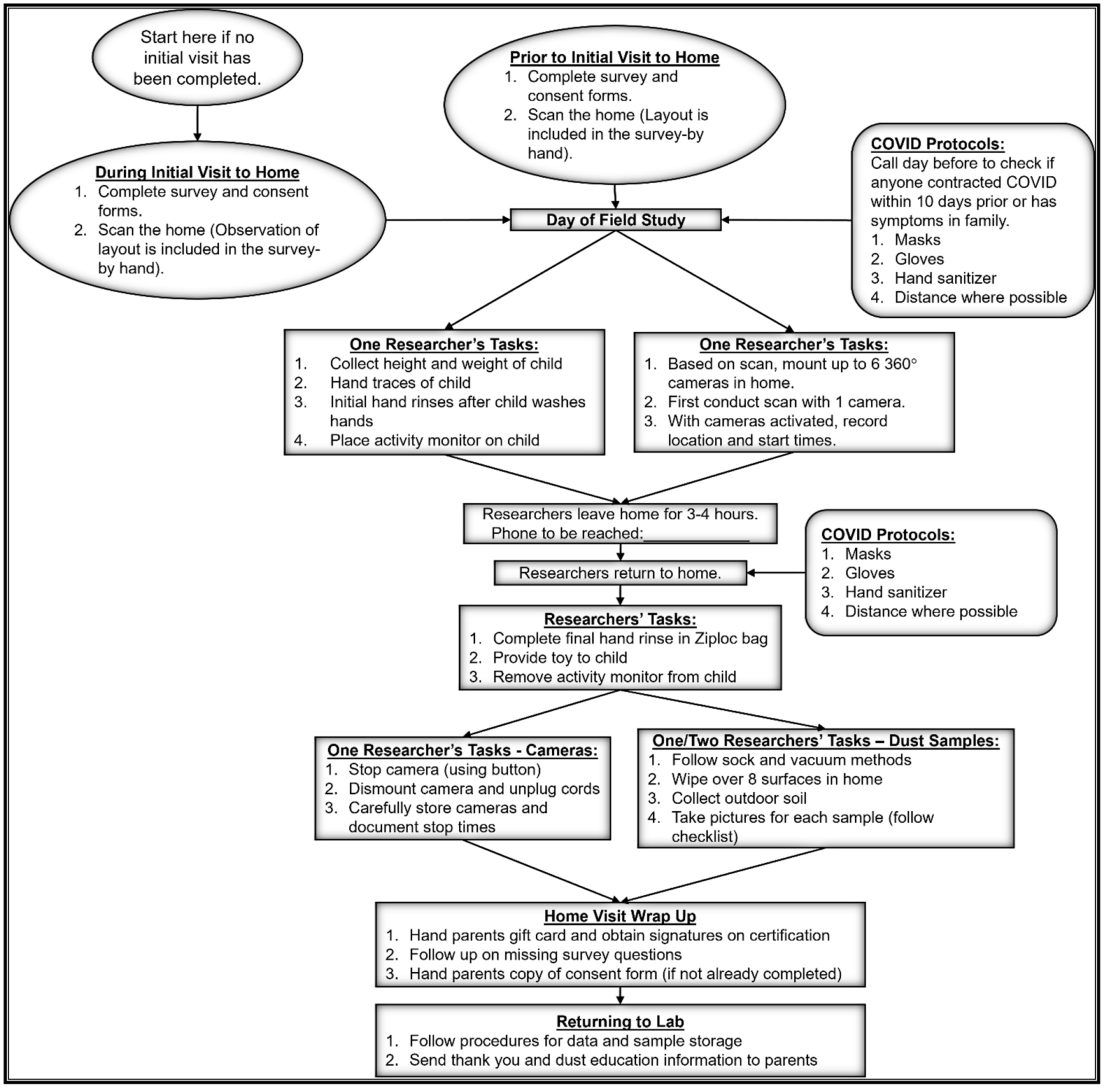
Field activities followed specific procedures for the collection of field data across the three regions to include survey collection (completion of home observation section in person), videotaping, hand rinses, dust and soil samples.

This section provides a summary of the sample collection process, with more details in subsequent sections. For consistency of data collection and quality assurance, researchers at the three collection sites (Arizona, Florida and North Carolina) followed field process and protocols for videotaping and dust/soil collection (Figure 2). Most participants who took part in the field data collection, completed the survey portion of the study prior to the in-home visits. If not, the survey and consent forms were completed on arrival to the participant’s home. Additionally, the observational section of the survey was completed, along with measurement of the room dimensions (length, width, and height) using a handheld digital laser. All rooms were measured unless the participants did not want us to enter an area (e.g., to not to disturb a family member). The child’s weight and height were then measured, followed by a wash of both hands (using the same soap across all study sites). Handwashing was followed by an initial hand rinse, collection of hand traces (explained further below), and an activity monitor was placed on the child. Cameras were mounted to elevated locations away from the child’s reach in up to six rooms of the home.

Researchers would then return in 3 to 4 h, where a final hand rinse was taken for the child and cameras were dismounted. Dust samples were then collected which included 8 surface wipes from various surfaces around the home, one vacuum sample, and two soil samples outside the home. Upon collection, all samples were immediately placed in their own zip-top bag to avoid cross contamination. For surface wipe and vacuum samples, researchers collected from rooms the child was more likely to play in but tried to get an assortment of surfaces representing varying loading possibilities in the home (e.g., dressers, windowsills, couches, top of fridge). For the vacuum sample, the main floor where the child spent most of the time was sampled. When possible, soil samples were taken from the back and the front of the home. Pictures were taken of all areas sampled. All samples were accompanied by sheets denoting the child alphanumeric code, time of sampling, researcher who collected the data and any other related data points. Participants also receive a thank you note and educational brochure (i.e., dust educational toolkit) and a link to the project website to track any updates to the educational tips and project outputs. The project website is located,^1^ and includes the dust educational toolkit.

All dust, soil, vacuum and hand rinse samples were processed and shipped to the Florida research team. Processing entails copying all of field data sheets for upload to Open Science Framework (OSF)^2^ before shipping, bagging, properly labeling samples, and ensuring rinse samples were sealed. All videos were uploaded to a password protected Google™ drive for access and processing by the North Carolina team. Field data sheets, and consent forms, were also copied and uploaded to OSP, and only shared with team members. Original files are stored in locked cabinets in the offices of the PI and CoPI’s at the three institutions.

### Collection and analysis of field samples (dust, vacuum, soil and hand rinse samples)

#### Overview of the collection of the surface dust samples (surfaces and hands)

The purpose of the child hand rinse samples was to obtain a measure of the dust loading on hands. Direct measurements of dust on children’s hands have traditionally been limited by the small mass of dust relative to the wipe used to draw the dust off the children’s hands, making it difficult to obtain a quantifiable measure of the mass of dust by gravimetric methods (e.g., difference in mass) (60, 61). Even when adhesive tape is used, the mass of dust/soil collected relative to the mass of the tape represents quantification challenges, and this method is not well suited for collecting dust/soil within skin folds and creases (62, 63). For studies focused on specific chemicals, the mass of dust can be bypassed through measurements of the contaminant mass (61, 64, 65). However, this approach is suitable for only the contaminant evaluated. It does not provide information about the mass of dust which is applicable to a broader range of contaminants.

**Figure 3:**
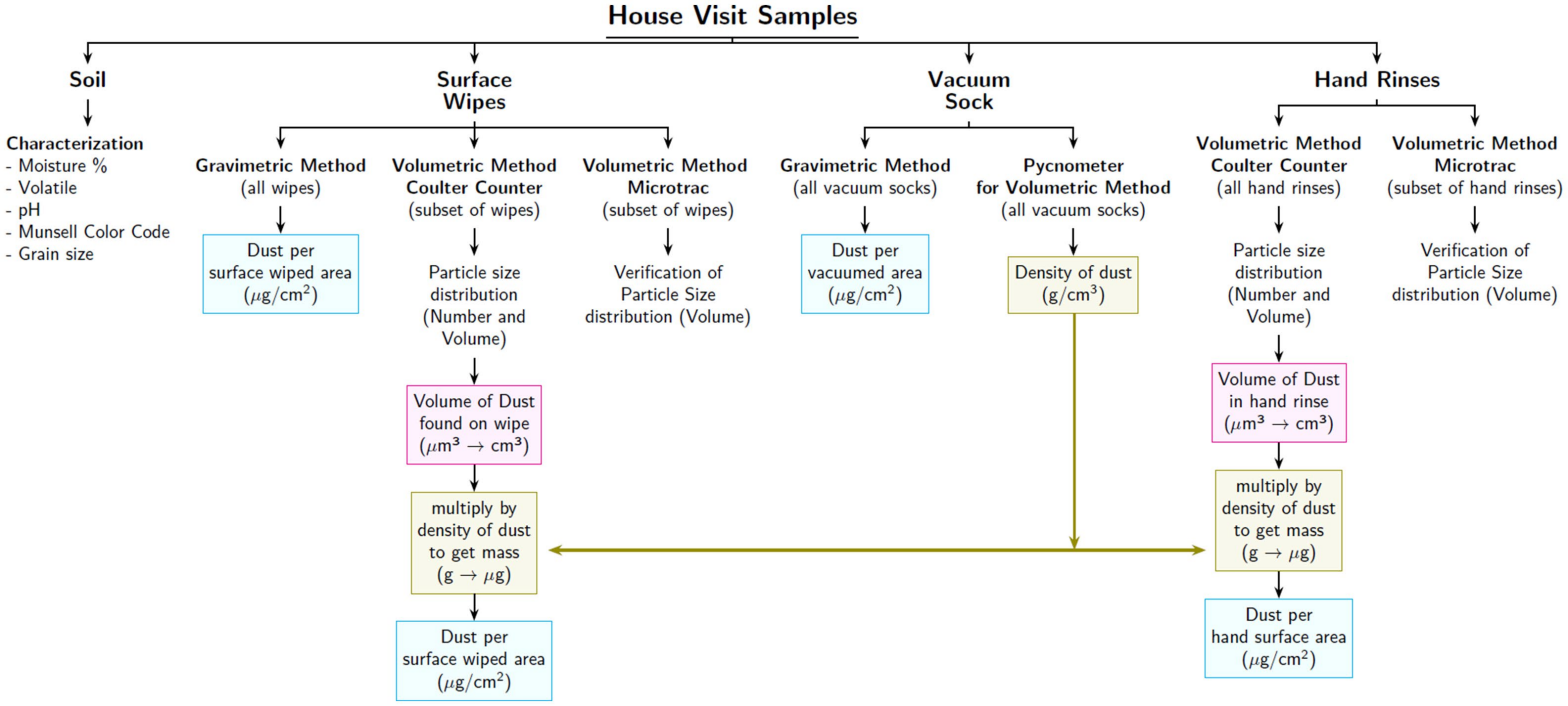
Display of how each sample was used for the analysis of household dust. All mass calculations were normalized by the surface area obtained through hand traces.

The current study utilized both the traditional gravimetric method and an innovative and highly sensitive volumetric method to measure dust mass per unit surface area. For larger dust samples (most surface wipes and all vacuum samples), the masses were determined gravimetrically by mass difference before and after wiping or vacuuming a known area. For smaller dust samples (all child hand rinses and a small proportion of surface wipes with masses below gravimetric detection limits) the new volumetric method was used. The volumetric method was based upon the fact that the mass of the dust, *M*, is the product of the volume, *V*, and density, *ρ*, such that *M* = *V* × *ρ*, where *V* is the volume of the dust collected from the surface or from a child’s hand after a set activity period, and *ρ* is the density of the dust as measured using a pycnometer (Figure 3). All measures of *M* were normalized by surface area, *A*. All measurements of mass were conducted using the same weighing scale (Mettler Toledo ME204TE) with a sensitivity of 0.1 mg.

#### Sample collection for gravimetric measurements of mass

Two sets of samples were processed gravimetrically. These included surface wipe samples (n = 8 per home) and vacuum samples (*n* = 1 per home). Samples were collected using dust free wipes marketed for clean room use (Kimberly-Clark Kimtech 33,330 Pure Disposable Wiper with W4 Dry) and were cut to 5 cm by 5 cm size using gloves and dust free scissors and then weighed. To control for surface area, stencils with a fixed surface area were used (25 × 25 cm, 25 × 15 cm, 25 × 10 cm, and 25 × 5 cm), selected based on the maximum option that could fit in the chosen sample location. Standard methods were used to collect the wipes (66). In brief, this method involved wiping the surface within the stencil in a descending back-and-forth motion for 30 s, with the goal of collecting a uniform and representative sample across the entire face of the wipe. Once done, the wipe was folded (dust toward the inside) to continue the wiping for an additional 30 s. After completing the wiping process, the wipe was placed back into its corresponding zip-top bag and kept at room temperature until reweighing. All wiping procedures required personnel to wear gloves. All wipe samples for mass (pre and post wiping) were measured using the same scale. Surface wipe masses were normalized by the surface area corresponding to the stencil used.

Vacuum samples were processed in a similar fashion by focusing on an area which was then measured for size and timed. The main purpose of the vacuum samples was to obtain enough dust (about 1 gram) such that the density measurements could be made. Vacuum samples were collected using an electric vacuum cleaner (Hoover Commercial CH30000) fitted with a pre-weighed dust collection sock (Midwest Filtration LLC, x-cell 100) (67, 68). A dust collection sock is an insert designed for inclusion in the vacuum tube intended to capture dust within a smaller volume and within a containment that can be pre-weighed. As a result, the vacuum sock, in addition to collecting enough dust for the density measurement, was also used to estimate the amount of dust on a floor. The vacuum sock samples were collected from the floor in areas where children spend a significant amount of time according to their parents/guardians (69). The type of floor material (e.g., carpet, floor, tile) was documented, the area was demarcated using a heavy chain, photographed, and measured using a laser ruler prior to vacuuming. The area was vacuumed for a minimum of 5 min using a similar back and forth motion as the surface wipes, ensuring that all of the area marked by the chain was vacuumed. After vacuuming, the sock containing dust was immediately removed and sealed into a zip-top bag for return to the laboratory, where it was reweighed. The mass of sample collected was then normalized by the surface area of the floor that was vacuumed.

One vacuum sock per household is unlikely to represent potential exposure to dust given that homes are characterized by many types of surfaces. It is for this reason that the 8 surface wipe samples were collected per home for they were quicker and less disturbing to participants in the home. In addition, we prepared an architectural layout for each home that describes the sizes of rooms within the home along with the flooring type. By aggregating all homes analyzed within a region, we anticipate obtaining enough vacuum dust data by flooring type to be able to estimate the potential exposures by the child in different parts of the home.

#### Sample collection for volumetric measurements of mass

Volumetric measurements were made for two sets of samples: all child hand rinses and for surface wipes for which the dust mass was below the gravimetric detection limit. The volumetric measurements were based upon transferring the particles on the child’s hands or on the wipes to a liquid (isotone consisting of 9 g of NaCl per liter of Milli-Q water, filtered through a 0.2 μm filter). The volume of particles in these samples, *V*, was measured using a Coulter Counter (Multisizer 3 Beckman Coulter, Brea, CA, United States), which provided the number of particles per unit volume and the particle size distributions (70). The total volume of the particles in the isotone was then converted to mass by multiplying by the density of the dust which was captured from the vacuum sock samples.

An initial background rinse sample of the child’s hands, called the pre-rinse was collected by first washing the hands with soap (California Baby Super Sensitive Shampoo and Body Wash, USDA Certified) in the sink to begin the study with child clean hands. Prior to the pre-rinse, the child’s hands were traced unto 1 cm^2^ ruled paper to be later used for surface area calculations. During the rinsing procedure after the child washed their hands, a study team member held the top of the pre-labeled bag filled with 150 mL of isotone while the parent or guardian guided the child’s hands into the bag and proceeded to gently rub the child’s hands from the outside removing as much of the dust as possible. After washing, the child’s hands were wiped with a dust free wipe (full size Kimberly-Clark Kimtech 33,330 Pure Disposable Wiper with W4 Dry). At end of the study period and videotaping, the child’s hands were again rinsed in 150 mL of isotone contained in a pre-labeled zip-top bag for the post rinse. Each rinse was timed for 20 s. In both cases, the zip-top bag was closed, and placed in a plastic container for delivery back to the laboratory where the samples were batched and shipped for analysis.

Of the 800 surface wipe samples collected, 12 were below the detection limit and required analysis via volumetric methods. Surface wipes were placed inside a zip-top bag, with added 150 mL of isotone and rubbed for 5 min to transfer the particles from the wipe into the solution. The cumulative volume of the particles in this solution was also measured using the Coulter Counter.

All samples subjected to volumetric determination of mass were normalized by the corresponding surface areas. For the hand rinses, the children’s palmer hand tracings (both hands) were digitized using ImageJ software^3^ to obtain the surface area of the child’s palmar hands (71). For the surface wipes, as described for the gravimetric method, the mass was normalized by stencil area used for wiping.

To validate the volumetric approach, a control hand rinse (fixed amount dust [0.01 g of the Arizona Test Dust, from Powder Technology Inc., PTI], added into zip-top) will be subjected to the volumetric method for comparison. Additionally, surface wipes for which gravimetric data were obtained will also be subjected to the volumetric method and compared. Such results will provide an estimate for the expected accuracy of the volumetric method of analysis.

#### Measurements of dust density

Vacuum samples were used for measurements of dust density (*ρ*). To calculate the density of dust, pycnometers (1 mL in size) were used according to the standard methods (72). Depending upon the mass of dust collected through the vacuum sock, pycnometer tests were run up to three times per home assuming sufficient mass available for analysis. All pycnometers were first calibrated using a pre-calibrated weighing scale (sensitivity of 0.1 mg). Each calibration consisted of the verification of each pycnometer’s volume, by filling with de-oxygenized Milli-Q water and calculating the mass of water added by difference. This mass of water was then multiplied by the corresponding density of water (given its temperature measurement near 20°C) to get the total volume of the pycnometer. After 10 replicates, the average value was calculated for the volume of each of the pycnometers.

##### Soil collection around the home and analysis

Two soil samples were collected per home, from both the front and back of the home, if possible. For apartment buildings, samples were preferably collected from the closest outdoor area with soil cover or indoor pots with soil if any. Samples were taken from the first few inches of the surface soils with stainless steel spoons and placed in zip-top bags. Soil samples are intended to be analyzed for texture and color based on a soil taxonomy system (73) coupled with general information available from the Natural Resources Conservation Service which provides general soil properties by region.

### Video-taping and videotranslation methodologies

#### Video-taping procedures

A novel methodology for videotaping that may have less impact on altering behavior was adopted in this study. Across the 100 homes in the three regions, small 360° lens tracking cameras were mounted to record the subject’s (i.e., the child’s) activities throughout rooms of the home without the presence of researchers. The 360° (Model: Insta 360 One X2 - Super 5.7 K Dual-Lens 360 Camera +4 K Wide Angle 60FPS) camera is designed to capture the full scan of a room. The camera integrates artificial intelligence (AI) features to track motions of subjects. This tracking feature allows the subject to be deep-tracked when highlighted allowing for a more focused video to be exported. Deep-tracking is an advanced camera feature for improved subject recognition to allow a subject to stay center-framed. This helps the observer to have a clearer and up-close view of the subject. Video-footage from 360° cameras can suffer some dark quality and lighting issues; however, the deep tracking feature attempts to limit this. The 360° cameras record in segments of up to 90 min before restarting a new segment. Video clips from various cameras in the home will be merged for one consecutive video of the subject over the 3 to 4-h period, providing a natural, sequential occurrence of events. To maximize video capture of the subject, up to six cameras were mounted in the home across rooms (excluding bathrooms). Traditionally for past videotaping studies that involve the collection of MLATS, children have been followed with handheld cameras (38, 42, 74, 75) which may influence or distract the subject, especially in small spaces, causing the MLATS to misrepresent true child behavior.

Cameras were mounted using several methods, including tripods, clamp mounts, and in many cases using medium strength mounting strips (brand: Command Strips). The cameras were mounted at 5–7-foot heights (depending on height and access in homes) at angles to maximize capture when the mounting strips were used. Tripod heights are close to six feet. Researchers experimented with options less likely to damage walls or remove paints, prevent interference by residents and easy to mount. Housing layout, including furniture location, wall mounting, and wall angles, also affected the preferred mounting type.

#### Videotranslation to produce micro-level activity time series (MLATS)

**Figure 4:**
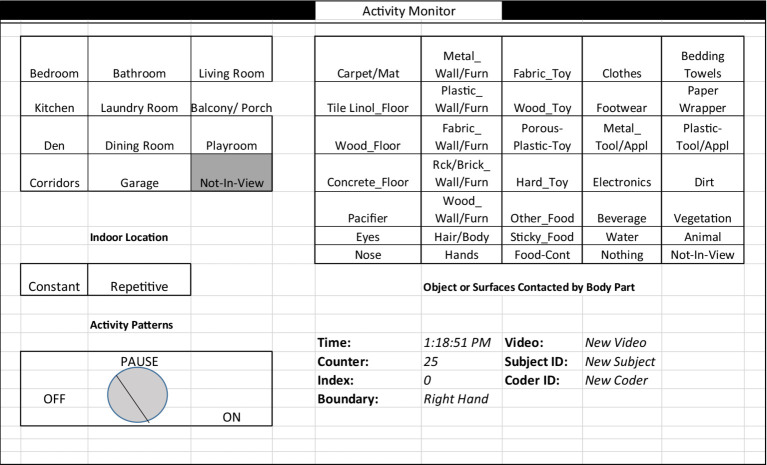
Activity virtual timing device palette for videotranslation. The palette includes locations commonly found in homes, object categories, an on/off button for recording time durations, activity patterns that note general exertion levels, and activity type, whether constant or repetitive. Shown here is the palette for translating mouthing events as it includes “hands” as an option, whereas for the hand palette, the word “Hands” is replaced with “Mouth” as an option for an object that can be touched.

The Activity Virtual Timing Device (AVTD) is used to translate the contact activities of both hands and mouth according to our previously established protocols (38–40). These protocols outline the process for videotranslation training on the software, and use of the palette once video-translation of the mouth and the left and right hand begin. While observing a video following a child, project personnel selected the appropriate choice within the AVTD categories, shown in grids to simplify translation efforts (Figure 4). During each observation, viewers tracked either the left hand, right hand or the mouth, where each change in location or object being touched (or no longer touched) required a new selection from the palette. A clock was activated during the translation to measure the duration of each unique combination of child activity. The translation creates text files of sequential activities for the body parts of hand and mouth important for this project on ingestion exposures (Table 1). Activities of the hands were used to compute dust loading to the hands and transfer to the mouth, which could then be correlated with the hand rinse data collected before and after the activity tracking.

**Table 1:** Example text file generated from VideoTraq (right hand observed): micro-level activity time series (MLATS) are created from the VideoTraq software indicating the activity type, location, object/surface the child is engaged in for a time period and are output as a text file.

Activity type	Location	Object/Surface	Duration (Seconds)
Constant	Bedroom	Hard_Toy	15
Constant	Bedroom	Nothing	5
Constant	Bedroom	Hard_Toy	30
Repetitive	Kitchen	Clothing	12
Constant	Kitchen	Nothing	2
Constant	Kitchen	Sticky_Food	45

These text files can be processed for frequency and durations of contacts patterns, but also be used directly in model that use the sequential data to assign mechanisms of contact and estimate loadings.

Most of the categories on the AVTD palette were developed in previous studies to represent likely surfaces found in homes. We added new key objects and surface categories of further interest. For example, we added “pacifiers” since young children often place in their mouth, and “electronics” which have become more commonplace for children to touch in their younger years. Pacifier use has been associated with risks of certain bacterial colonies and illness for an infant or child, especially when not sanitized (76), and may also have higher levels of dust if dropped on floors routinely. The addition of the “electronics” category can benefit children and health studies interested in time spent with electronics in today’s modern world. We pre-classified all objects/surfaces observed in homes across the regions by pre-watching all videos. Objects that fitted into multiple categories were associated with the option that provided the highest exposure risk in order to be more protective against possible adverse health outcomes. The palette contained categories for locations commonly found in homes and included an option for when the child was not in view. The contact pattern with an object was also described as constant or repetitive, where repetitive actions included things like crawling or bouncing a ball, and where rapid changes between touching an object and touching nothing were not easily captured.

For this study, 300 to 400 h of micro-activity patterns will be processed for young children in sequential second-by-second lines of activity for the left and right hands and mouth. These MLATS sequences will be used directly in the model for estimation of dust ingestion for these children. Frequency and duration of contact activities will be extracted and analyzed for differences by US EPA age groups, race, income, region and built environment using non-parametric methods (34, 74, 77, 78). Likewise, dust loading, hand rinse data, and variables from the survey will be evaluated for associations with US EPA child age groups, race, income, regions, built environment, seasons, and soil characteristics (moisture, particle size distribution) via simple and multiple regression.

### Use of activity trackers on children

Researchers also placed an activity tracker (Model Fitness Tracker HR by Brilliant House) on the children. The activity tracker is a wristband that uses a three-axis accelerometer, where each acceleration of the human body is converted into steps (±2% error). The activity band was put on a child’s wrist, arm or leg (the smaller the child, the more likely to be placed on leg), and covered with elastic workout bands to prevent accidental or purposeful damage or movement. This helped track children’s steps and movement during the videotaping period, allowing for the opportunity to compare the activity levels from videotaping and videotranslation data to denote resting, walking and running designations/categories in a MLATS translation.

## Results (anticipated)

### Child-specific aggregate cumulative human exposure and dose model framework

This project will apply the Child-Specific Aggregate Cumulative Human Exposure and Dose (CACHED) model framework to estimate dust/soil ingestion for young children, which is an agent-based model that uses MLATS (43) which, when combined with environmental concentrations (e.g., dust loading on a surface) and exposure factors (e.g., soil-skin adherence, contact area), can compute realistic estimates of dermal loading, and subsequent non-dietary ingestion exposure (43, 79). Another feature of CACHED is the ability to run Monte Carlo simulations (i.e., repeated model runs using random sampling from parametric distributions to incorporate variability and uncertainty in inputs), which allows the user to create “virtual” children based on expected characteristics and behaviors. This increases the sample size, and provides more power for statistical analyses. The number of simulations depends on where the model reaches stability in its results. The dermal and non-dietary ingestion exposure modules construct, equations, and output options were developed in accordance with exposure and contact boundary definitions presented in Zartarian et al. (80) and Canales and Leckie (43). CACHED also contains additional modules for inhalation exposure, dermal absorption and physiologically-based pharmacokinetic modules to estimated cumulative (i.e., multiple chemicals) and aggregate exposures (i.e., multiple exposure routes) and dose while accounting for children’s age and gender (81). CACHED has been previously used to simulate pesticide exposure for a population of farmworker children (45, 81). In that study, the CACHED model predicted pesticide metabolites in urine that were not significantly different from corresponding measurements, and the estimates indicated that non-dietary ingestion exposure was the primary route. This indicated the reliability and value of the CACHED modeling approach. Similarly, this approach was adapted for estimating viral loading on hands that was not significantly different from measurements in an office setting (44).

**Figure 5:**
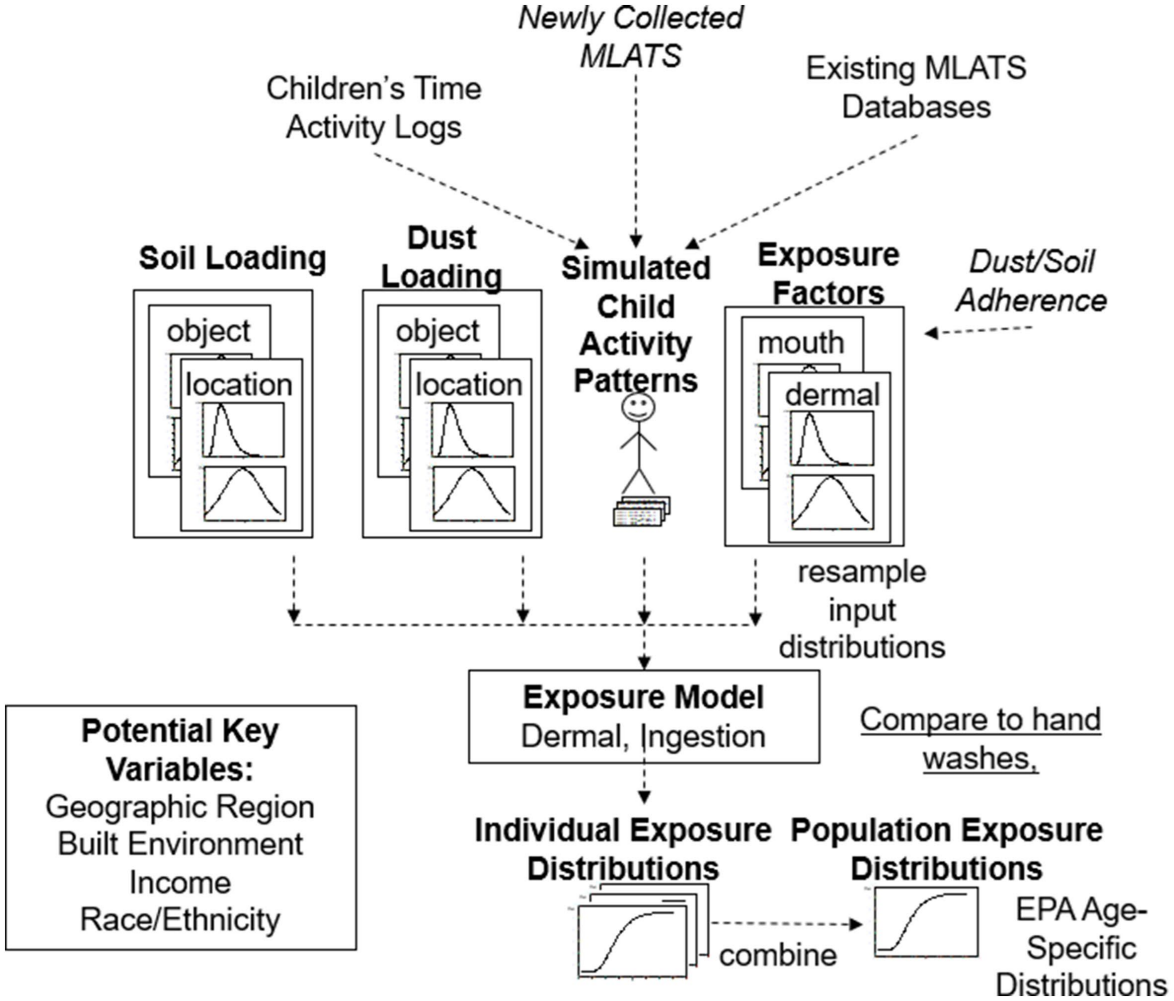
Newly collected MLATS data from this indoor study and previous indoor and outdoor studies were used to simulate child activity patterns for use in our modified exposure model to produce individual dermal and ingestion exposure distributions. Individual distributions of exposures can then be combined into a population estimate, as was done in CACHED. Soil loading and dust loading collected from the homes were also used in the exposure model [Figure Adapted from Beamer et al. (31)].

For non-dietary ingestion modeling and the calculation of dust/soil ingestion rates in this study, the model estimates ingestion based on contact of objects and surfaces with the mouth (e.g., toys, hands). Since one of the primary exposure mechanisms is dust/soil ingestion from mouthing of hands, it is important to know the loading of dust/soil on the hands, estimated through the dermal exposure module of the CACHED modeling framework. Dermal loadings can then be used to develop parametric or empirical distributions that then serve as inputs for the non-dietary ingestion exposure simulations for hand-to-mouth contacts. In using CACHED, individuals’ sequential MLATS (i.e., mouth contacts with contaminated surfaces, placing hands or fingers in the mouth, mouthing objects like pacifiers or toys) form the backbone of the model and are combined with dust/soil loading and exposure factor distributions to estimate children’s daily dust/soil ingestion rates from data gathered in this study and other previous studies (Figure 5). For example, distributions for dust loading on different surfaces types will be developed from the samples collected and analyzed in the lab from the 100 homes in this study. This will be combined with data from other studies in the field for dust loadings and dust loading distributions (18, 74, 82, 83).

The goals of the current project are to utilize the CACHED framework to estimate daily indoor dust ingestion and outdoor soil ingestion for children ages 6 months to 6 years by US EPA age categories. We are developing and coding an additional module for CACHED that will combine time activity diaries (i.e., sequence of time indoors and outdoors) with MLATS collected for children indoors versus outdoors. The time activity diaries will come from information collected from this study and from other sources including American Time Use Survey (ATUS) and Consolidated Human Activity Database (CHAD) (84, 85). The MLATS will come from multiple studies conducted by the study team (38, 45, 74, 75, 78, 86). Most of these studies are primarily focused on children’ outdoors, hence the study team collected additional videography and MLATS for young children in the indoor environment. Supplementary Table 1 is a list studies and variables that will provide the model with additional critical data to calculate both indoor dust ingestion and outdoor soil ingestion. The activity data and surface loadings from this study will be used as inputs for the model, while the hand surface loading will be used to verify the dermal exposure simulations and assumptions.

Within the CACHED framework, each contact is assumed to be an independent event, and new values are sampled from each of the input parameter distributions reflecting the potential variability and uncertainty associated with each contact. There are limitations in the model therefore to be able to accurately reflect loading and off-loading dynamics, and even maximum loadings on hands. The dermal exposure estimates of hand-loadings, will be compared with our hand rinses as part of our iterative modeling process to help adjust assumptions in the model. As an example, for young children where pacifier use is very common, there is little known on the loading, offloading and washing habits of parents. As a result, assumptions will need to be made for calculations and loading and removal dynamics per contact event. CACHED exposure modules were originally coded in S-Plus but will be reprogrammed in RStudio 2022.07.1 with R version 4.2.1 (2022-06-23 ucrt) (87).

## Discussion

This study can greatly contribute to the limited data available to quantify dust/soil ingestion rates, especially for young children aged 6 months to 6 years. Additionally, our study will provide increased information on Black and Hispanic populations. The use of newer and more sensitive methodologies can improve the accuracy of risk assessments based on the quality of activity data, especially the dust ingestion rates in homes. Improved quantification of dust loadings on surfaces through volumetric methods will allow for lower detection levels for mass quantification, improving the accuracy of measuring dust loading on hands and, therefore, dust ingestion rates, especially in areas characterized by low levels of dust. To reduce the risk of exposure to contaminants, it is critical to investigate dust/soil ingestion rates, particularly in young children aged 6 months to 6 years, and to improve methodologies to obtain more accurate data on dust/soil ingestion rates.

There are some limitations to the methods used here. We used a limited dataset of children for the videotaping and dust collections, and we may have incorporated biases into the assumptions that were made regarding the accuracy of our population’s parameter distributions compared to the true population distribution. Convenience sampling can further introduce sampling bias such as oversampling one population versus the other and one group may have differential exposures. However, for this study the intention is to oversample some minority groups and look at behaviors and outcomes by sociodemographic variables. Other biases can be introduced unknown to researchers such as potentially participating parents exhibiting better/more intentional caregiving traits and home care traits. Some biases can be inherently harder decipher or measure. The use of Monte Carlo simulations to model “a child” can expand individual risk assessments to population assessments can address some biases and uncertainty (88–91).

In terms of the videotaping techniques and the ability to capture true behaviors, although children are less likely to be aware of the mounted cameras, parental behavior may still be altered. In addition, we captured only 3–4 h of activity for each child on a single day. Measuring dust loadings for every possible object that a child can touch, or mouth can be extremely time and cost intensive, and as such the dust loadings may vary across more than just the 8 surface wipe samples collected in this study. To address these limitations, more data can be collected by others to further add to the dataset for children’s behaviors, dust loadings and ultimately ingestion rates using similar field and data processing methodologies. In addition, soil collected around these homes may also not be representative of the dynamics that occur at other homes. Soil, collected from pots for example, could either have originated from soil around the area or likely from a store and will not represent a relationship to indoor track in dust.

There are other methodological limitations. For example, limitations of the hand rinse approach are that it does not consider parts of hands or fingers involved in contacting soil/dust surfaces or the end portions of fingers involved during mouthing activities. These limitations or assumptions made need to be acknowledged properly. For future studies, the volumetric approach can possibly be used to isolate dust from these parts of the hands by carefully rinsing sub portions of the hands.

Videotaping using multiple cameras also comes with challenges. Proper and advantageous placement of cameras may prove to be an important aspect for the collection of clear video-footage.

When mounting cameras, there were additional considerations that affected the placement, such as the availability of power outlets in homes. This proved to be a challenge, especially in older homes that tended to have fewer outlets. We avoided plugging into resident’s electrical power strips since electrical failure and loss of power to camera tended to occur more often when connected to them. Future studies can explore camera technologies that have longer battery power (battery could only run for about 80 min) or use of battery packs. This project can be used as a reference for future studies interested in applying this technology and methods in public and work environments to capture exposure activities.

### Other funded studies on soil/dust ingestion

There was a total of seven teams of researchers funded under EPA’s RFP focused on children’s exposure to dust/soil in 2021 (92). Researchers from Emory University (PI: Eri Saikawa) proposed targeted and non-targeted soil analysis to map lead (Pb) and other contaminant concentrations in a community of West Atlanta. Using the biokinetic approach, in a complementary and systematic approach, oral saliva, blood and urine biomarkers will be analyzed to estimate dust/soil ingestion for children. This group of researchers had previously found high levels of metalloids in the soil of this community that may not have been suitable for urban gardening and food consumption (93).

Florida International University researchers (PI: Natalia Soares) will use a non-targeted screening approach (liquid chromatography–high resolution mass spectrometry) to identify tracers of dust and soil ingestion, especially tracers that might be more accurate for warmer regions. The study will utilize information from children’s activity behaviors, and chemical analysis in food and urine, to refine the choice of tracer and to estimate dust/soil ingestion using the SHEDS-HT Model. The Stochastic Human Exposure and Dose Simulation Model for High Throughput (SHEDS HT) model, developed by EPA, facilitates the assessment and prioritization of chemicals for risk assessment (94).

Researchers from Johns Hopkins (PI: Keeve Nachman) will rely on macro and micro activities and tracer studies to address dust/soil ingestion rates for children. Specifically, artificial intelligence (AI) and computer-aided approaches to generate object and mouth frequency data for young children, while non-targeted analysis will be used to characterize unregulated organic fingerprints in environmental and biological samples for young children. Time activity diaries and telephone interviews aided the collection of macro activities. This group had previously used a meso-activity for farming activities with key consideration to address the potential for soil ingestion for farmworkers (95, 96).

New York University (PI: Karen E Adolph) will use robotic platform to measure surface transfer rates for dust, and video data to extract hand-to-mouth and hand-to-surface contact patterns for young children. In addition, chemical and physical analysis was conducted on dust samples. Transfer rates, activity patterns and dust analysis results will be compared across home characteristics, demographics and child age groups, which will be used in a modeling approach for dust ingestion rates. This group has previously published on the motor development for young children (97), where a child’s development may affect contact patterns and ultimately dust/soil ingestion.

A novel approach to study 80 possible tracer compounds found in household dust as tracers for dust/soil ingestion is proposed by the University of California Davis (PI: Deborah H Bennet). The study will make use of the ‘Limiting Tracer’ and ‘Best Tracer Methods’. Dust ingestion rates will be compared across four distinct regions and sociodemographic/health backgrounds (to include children with neural developmental outcomes). Urine analysis will be completed across the children and compounds were also analyzed in dust. This group had previously shown that house dust can be repository for semi-volatile and other chemicals, putting women and child at risk for exposure and adverse outcomes (98, 99).

University of Nevada (PI: Li Li) will use an integrative approach to examine dust/soil ingestion for children, by using a reverse dosimetry/structure equation model to determine from biological measures (23 biomarkers) the dust/soil ingestion rates for young children. Children’s touching and mouthing behaviors will be collected for use in the model. This group has previously published on mouthing-mediated ingestion using a mechanistical model, considering the physicochemical properties of chemicals and human activity (100).

In summary, these funded studies are utilizing modeling, tracer and biokinetic approaches or combinations to address dust/soil ingestion for young children, where some of their objectives and activities may change. The majority of funded studies will employ some type of children’s activity patterns (micro, meso, macro), collected through a variety of methods (observation, videotaping, survey) to compliment or facilitate the estimation of dust and soil ingestion for children. Sample sizes for participants vary but ranged between 30 to 450 participants depending on the type of data being collected. Samples sizes (e.g., dust or urine samples) varied depending on how many samples are being collected on each participant. Most will address dust ingestion rates in the home. Many will also use a variety of lab techniques to find suitable and ubiquitous tracers that can be used in future studies to address dust/soil ingestion and exposure to varied contaminants.

Grantees of this EPA RFP met in early December 2022 at EPA Research Triangle Institute in Raleigh Durham to present their planned work and to look at complementary areas of research and collaborative opportunities. Historically, this will be one the greatest focused efforts and funding support placed on the collection of young children’s dust/soil ingestion rate nationally and concurrently to date. During this meeting, researchers expressed an expectation of a future wealth of information and new approaches to be generated from these studies, with clear indication of challenges, limitations, and suggestions for continued research.

## Conclusion

Dust/soil ingestion rates are critical factors for estimating exposure to a multitude of contaminants that are found in dust/soil particles. These ingestion rates are, in fact, one of the most uncertain factors in exposures and health risk estimates. Improved policies and guidance for exposure to a variety of contaminants found in dust (e.g., metals, PAHs, phthalates, PFAS) are needed. Dust/soil ingestion rates are utilized in decisions regarding the registration of products (e.g., pesticides), and chemicals under the Frank R. Lautenberg Chemical Safety Act for the 21st Century and Superfund Law (101). This project adds to the dataset of dust/soil ingestion rates for children to help improve health risk estimates for this vulnerable population and provides a model approach that integrates and harmonizes existing and new data in the field. Better estimates of exposures will facilitate improved health risk estimates and targeted strategies for the protection of children and families in addressing dust and soil exposures. This project also explores the variation in dust levels and children’s activities by geographic region, which may vary by housing stock, soil type, and climate and whether homes are situated in rural, suburban, or urban settings. Importantly, this project will highlight the importance of dust and soil ingestion for children and develop tools (website, educational tools and tips) that can be used to empower families to reduce the risk of exposure. We will be following children in their normal daily activities and attempting as much as possible to reduce interference. There will be opportunities to compare the data collected on this project to data being collected on dust/soil ingestion from six other teams across the country and address any limitations and data gaps in the future. Collectively, the methods described in detail here, along with the approaches taken by the six other funded research groups, will contribute toward a better understanding of children’s exposures to dust/soil in the home. This information can be translated to provide recommendations to families and inform policies aimed at improving child health. The results from these studies are expected to inform the US EPA during their review and possible revision of its guidance on recommended soil and dust ingestion rates for the four different age categories of children considered in our research.

## Data availability statement

The original contributions presented in the study are included in the article/Supplementary material, further inquiries can be directed to the corresponding author/s.

## Ethics statement

The studies involving humans were approved by North Carolina Agricultural and Technical State University Institutional Human Subjects Review Board. The studies were conducted in accordance with the local legislation and institutional requirements. Written informed consent for participation in this study was provided by the participants' legal guardians/next of kin.

## Author contributions

AF: Conceptualization, Data curation, Formal analysis, Funding acquisition, Investigation, Methodology, Project administration, Resources, Software, Supervision, Validation, Writing – original draft, Writing – review & editing. FA: Data curation, Formal analysis, Investigation, Methodology, Software, Supervision, Writing – original draft, Writing – review & editing. HS-G: Conceptualization, Data curation, Formal analysis, Funding acquisition, Investigation, Methodology, Project administration, Resources, Software, Supervision, Validation, Writing – original draft, Writing – review & editing. EO-G: Methodology, Validation, Writing – original draft, Writing – review & editing. CF-M: Data curation, Formal analysis, Investigation, Methodology, Software, Supervision, Writing – original draft, Writing – review & editing. MG: Conceptualization, Data curation, Writing – original draft, Writing – review & editing. JH: Investigation, Methodology, Software, Writing – original draft, Writing – review & editing. OO: Data curation, Methodology, Software, Supervision, Writing – original draft, Writing – review & editing. PB: Conceptualization, Data curation, Formal analysis, Funding acquisition, Investigation, Methodology, Project administration, Resources, Software, Supervision, Validation, Writing – original draft, Writing – review & editing.

## Footnotes

^1^https://www.dirt-child.com

^2^www.osf.io

^3^https://imagej.net/ij/
