# Supplementary material for: Methodologies for the collection of parameters to estimate dust/soil ingestion for young children
Source: Front Public Health. 2024 Jun 26;12:1357346. doi: 10.3389/fpubh.2024.1357346 (PMC11234889; doi:10.3389/fpubh.2024.1357346)
Supplement: Supplementary file 1 [file Table_1.docx]

Supplemental Table 1: Existing Datasets for Variables Related to Estimating Soil/Dust Ingestion for Young Children 6 Months to 6 Years and to Supplement Data Gathered for Indoor Dust Ingestion for Modeling Efforts. Data is used from the literature to supplement the needed parameters needed in the model to estimate soil/dust ingestion rates for young children.

| **Dataset** | **Authors/**  **Study** | **Methodologies** | **Geography/**  **Location/**  **Setting** | **Age Groups/**  **Homes (Relevant to this Study)** |
| --- | --- | --- | --- | --- |
| **Soil/Dust Loading** | | | | |
| 1. Soil/Dust Loading on Surfaces | Cho et al., 2006 [1] Barrio-Parra et al., 2018 [2], Edwards et al., 1998 [3]  Ozkaynak et al., 2011 [4] | Surface Wipes/Vacuums  Dust Loading Distributions | New Jersey, Spain | Homes, Apartment |
| 1. Soil/Dust Loading on Foods/Consumption Patterns | ***NA: Not Considered in this modeling effort-cross contamination can be considered*** | | | |
| 1. Soil/Dust Loading on Hands (adherence) | Ferguson et al., 2009, 2020 [5,6], Choate et al, 2016 [7], Kissel et al., 1996 [8], Holmes et al., 1999 [9]  Ferguson et al., 2020ª^,b^ [6,10] | Chamber Studies/Hand Press Trials/Rinse Studies/Wipes | Lab Based, Four Beaches in Gulf of Mexico, Tidal Flats, Recreational Areas | Controlled, 1 yr. to 6 yrs. |
| **Removal Efficiencies** | | | | |
| Removal Efficiency from Hands, Object/Surfaces | Beamer et al., 2008 [11] | Observation, Distribution | California |  |
| **Contact Frequencies/Duration** | | | | |
| Hand/Object to Mouth Contact/Frequency/Durations | Beamer et al., 2008 [11]; Xue et al., 2010 [12]; AuYeung et al., 2005 [13]; AuYeung et al., 2006 [14]; Beamer et al., 2012 [15]; Tsou et al., 2018 [16], Black et al., 2005 [17], Ferguson et al., 2021 [18] | Videotaping-Video-Translations/  Observation/  Surveys | California, US Mexico Border and elsewhere, Gulf Regions | Range in age across all studies is 6 months to 6 years months, |
| **Time Spent Data** | | | | |
| Time Spent In Location/Yearly | Tandon et al., 2013 [19]; Matz et al., 2014 [20], Silver et al., 1994 [21], McCurdy et al. 2000 [ 22] | Time Activity Log/Model of long-term exposure | Outdoor/Indoor at Child Care; United States, Canada, CHADS Multi-State | All ages, 3-5 years, 5-6 yrs. |
| **Surface Areas** | | | | |
| Hands (Full and Fractional Area of Contact) | Perone et al., 2021 [23], AuYeung et al., 2008 [24], Du Bois and Du Bois, 1989 [25] | Tracings, VideoTranslation, Algorithms | Beaches in Gulf of Mexico, California, Applied Anywhere | 1 Yr., to 6 Years, All child ages |

Note: Any sociodemographic, geographic, seasonal or age group data will be recognized in datasets

References

1. Cho S-H, Reponen T, Bernstein DI, Olds R, Levin L, Liu X, et al. The effect of home characteristics on dust antigen concentrations and loads in homes. Sci Total Environ. 2006.

2. Barrio-Parra F, De Miguel E, Lázaro-Navas S, Gómez A, Izquierdo M. Indoor Dust Metal Loadings: A Human Health Risk Assessment. Expo Health. 2018;10:41–50.

3. Edwards RD, Yurkow EJ, Lioy PJ. Seasonal deposition of housedusts onto household surfaces. Science of The Total Environment [Internet]. 1998;224:69–80. Available from: https://www.sciencedirect.com/science/article/pii/S0048969798003489

4. Ozkaynak H, Xue J, Zartarian VG, Glen G, Smith L. Modeled estimates of soil and dust ingestion rates for children. Risk Anal. 2011;31:592–608.

5. Ferguson A, Bursac Z, Coleman S, Johnson W. Comparisons of computer-controlled chamber measurements for soil-skin adherence from aluminum and carpet surfaces,. Environ Res. 2009;109:207–14.

6. Ferguson A, Dwivedi AK, Ehindero E, Adelabu F, Rattler K, Perone HR, et al. Soil, hand, and body adherence measures across four beach areas: Potential influence on exposure to oil spill chemicals. Int J Environ Res Public Health. 2020;17:1–20.

7. Choate LDM, Ranville JF, Bunge AL, Macalady DL. Dermally Adhered Soil: 1. Amount and particle-size distribution. Integr Environ Assess Manag. 2006;2:375–84.

8. Kissel JC, Richter KY, Fenske RA. Field measurement of dermal soil loading attributable to various activities: Implications for exposure assessment. Risk Analysis. 1996;

9. Holmes KK, Shirai JH, Richter KY, Kissel JC. Field measurement of dermal soil loadings in occupational and recreational activities. Environ Res. 1999;80:148–57.

10. Ferguson A, Rattler K, Perone H, Dwivedi AK, Obeng-Gyasi E, Mena KD, et al. Soil–skin adherence measures from hand press trials in a Gulf study of exposures. J Expo Sci Environ Epidemiol [Internet]. 2021;31:158–69. Available from: https://doi.org/10.1038/s41370-020-00269-2

11. Beamer P, Key ME, Ferguson AC, Canales RA, Auyeung W, Leckie JO. Quantified activity pattern data from 6 to 27-month-old farmworker children for use in exposure assessment. Environ Res. 2008;108:239–46.

12. Xue J, Zartarian V, Tulve N, Moya J, Freeman N, Auyeung W, et al. A meta-analysis of children’s object-to-mouth frequency data for estimating non-dietary ingestion exposure. J Expo Sci Environ Epidemiol [Internet]. 2010;20:536–45. Available from: https://doi.org/10.1038/jes.2009.42

13. Auyeung W, Canales RA, Beamer P, Ferguson AC. Young Children ’ s Mouthing Behavior : An Observational Study via Videotaping in a Primarily Outdoor Residential Setting Young Children ’ s Mouthing Behavior : An Observational Study via Videotaping in a Primarily Outdoor Residential. 2005;

14. AuYeung W, Canales RA, Beamer P, Ferguson AC, Leckie JO. Young children’s hand contact activities: An observational study via videotaping in primarily outdoor residential settings. J Expo Sci Environ Epidemiol. 2006;16:434–46.

15. Beamer PI, Luik CE, Canales RA, Leckie JO. Quantified outdoor micro-activity data for children aged 7 – 12-years old. 2012;82–92.

16. Tsou M-C, Özkaynak H, Beamer P, Dang W, Hsi H-C, Jiang C-B, et al. Mouthing activity data for children age 3 to <6 years old and fraction of hand  area mouthed for children age <6 years old in Taiwan. J Expo Sci Environ Epidemiol. 2018;28:182–92.

17. Black K, Shalat SL, Freeman NCG, Jimenez M, Donnelly KC, Calvin JA. Children ’ s mouthing and food-handling behavior in an agricultural community on the US / Mexico border. 2005;244–51.

18. Ferguson A, Dwivedi A, Adelabu F, Ehindero E, Lamssali M, Obeng-Gyasi E, et al. Quantified activity patterns for young children in beach environments relevant for exposure to contaminants. Int J Environ Res Public Health. 2021;18:1–19.

19. Tandon PS, Saelens BE, Zhou C, Kerr J, Christakis DA. Indoor versus outdoor time in preschoolers at child care. Am J Prev Med. 2013;44:85–8.

20. Matz CJ, Stieb DM, Davis K, Egyed M, Rose A, Chou B, et al. Effects of age, season, gender and urban-rural status on time-activity: Canadian human activity pattern survey 2 (CHAPS 2). Int J Environ Res Public Health. 2014;11:2108–24.

21. Silvers A, Thomas Fl~rence B, Daniel ~, Rourke L, Hrimo$ RJ. How Children Spend Their Time: A Sample Survey for Use in Exposure and Risk Assessments. Risk Analysis. 1994.

22. McCurdy T, Glen G, Smith L, Lakkadi Y. The National Exposure Research Laboratory’s Consolidated Human Activity Database. J Expo Anal Environ Epidemiol. 2000;10:566–78.

23. Perone H, Rattler K, Ferguson A, Mena K, Solo-Gabriele H. Review of methods to determine hand surface area of children less than six years old: a case study. Environ Geochem Health. 2021;43.

24. AuYeung W, Canales RA, Leckie JO. The fraction of total hand surface area involved in young children’s outdoor hand-to-object contacts. Environ Res. 2008;108:294–9.

25. Du Bois D, Du Bois EF. A formula to estimate the approximate surface area if height and weight be known.  1916. Nutrition. 1989;5:303.
